# Toxical Effects of Hydrate of Chloral When Persistently Used as a Hypnotic, and Fatal Results of Large Doses

**Published:** 1872-01

**Authors:** N. R. Smith

**Affiliations:** Baltimore.


### SELECTED ARTICLES.

---

### ARTICLE V.

### *Toxical Effects of Hydrate of Chloral when Persistently Used as a Hypnotic, and Fatal Results of Large Doses.*

BY N. R. SMITH, M. D., BALTIMORE.

In February last a medical friend, long retired from practice, called on me for advice in regard to a singular affection of the fingers of both hands, attended with desquamation of the cuticle and superficial ulceration, especially about the borders of the nails. It was attended with pain and much morbid sensibility to touch. It was also associated with some acceleration of pulse and general *malaise*. He visited me daily for some ten days, when by the use of astringent lotions and mild digestive ointment, the local affection was overcome. He informed me that he had been taking chloral in liberal doses, as a hypnotic, for four months. He expressed to me his conviction that the disease of his fingers had resulted from the use of that medicine.

Having never observed the agent to produce such a result, I was reluctant to believe that it was the case.

Some three weeks after the cure of the local affection, I was called to attend my friend in consultation with his family physician. We found him laboring under acute bronchitis in severe degree. His respiration was exceedingly embarrassed, and there was a high degree of hoarse mucous rale. The bronchial tubes were filling ; the pulse was about 140, and the action of the heart *extremely feeble.* By the treatment adopted, our object was to sustain the powers of life, which were rapidly failing, and to relieve the bronchial tubes of mucus. Our efforts, however, were unavailing. He died on the third day after I first saw him.

I scarcely, at the moment, entertained a suspicion that the use of chloral was concerned in producing the fatal malady of my friend, it being not at all uncommon for persons of his age (70) to succumb suddenly to such malady from ordinary causes.

Some three weeks later I accidentally met a medical friend, who expressed pleasure at the meeting, as he wished to consult me in relation to a singular affection under which his daughter, a young lady twenty-two years of age, was suffering. He described precisely the affections of the integuments of the fingers which had occurred in the case described above—erythematous inflammation, desquamation, and ulceration around the border of the nails.

Struck with the resemblance which her malady bore to that of my friend, Dr. C——, I inquired if she had been taking chloral. He replied that she had used it as a hypnotic a month, every night, and that he had suspected that article to be the cause of her disease.

The young lady was not suffering constitutionally at that time; but about ten days after I was called to see her. I found her extremely ill. There was universal anasarca. The action of the heart was exceedingly feeble, the pulse 140, and extremely weak. Her respiration was much embarrassed, and the recumbent posture was impossible. Procuring some of the urinary secretion, I tested it with my nitric acid, and discovered a notable quantity of albumen.

I was very apprehensive of a fatal result, but immediately prescribed stimulants and diuretics, digitalis being the constituent most relied upon.

On visiting the patient, after an interval of a day, I was much surprised and gratified to find her greatly improved. Her pulse had been reduced to 90, and was greatly improved in tone. The kidneys had acted freely, and the anasarca had much abated.

Having been myself confined by illness, I did not again see her. On meeting her father some three weeks later, I was gratified to learn that she had entirely recovered.

I have knowledge of two other cases in which the same affections of the fingers resulted from the use of chloral.

Within the last ten days two deaths have occurred in Baltimore, manifestly from the toxæmia caused by an overdose of chloral. The subject of one of these accidents had been under the care of an irregular physician, and by his advice had taken chloral in ordinary doses for the relief of a painful neuralgic affection of the neck.

After the medical attendant had discontinued his visits, the patient persisted in the use of the hydrate, taking it, as I was informed by his brother, in doses of not less than half a drachm. On the day of his death he was know to have purchased three drachms of the article. How much he took during the day is unknown. In the evening he retired to his chamber, and in about twenty minutes after he was found dead beside his bed. He was undressed, and the bedclothes were turned down, but the bed was undisturbed, and it was manifest that death had arrested him at the moment that he was prepared to step into bed. The coal-oil lamp which he used was extinguished, but the glass chimney was still hot. The glass from which he had taken the chloral stood on a small table near the head of the bed, and in it were a few drops of the medicine, recognized by his brother by taste and smell. There can be no doubt, therefore, that he fell almost instantly dead from the effect of the poison.

Another instance of almost equally sudden death has recently occurred in this community. The fact is generally known, but I am not authorized to name the individual. he had been laboring under a painful affection of the head, and was attended by a homœopathic physician. On the evening of the night of his death he had a hypodermic injection of morphine practiced upon him, probably in ordinary quantity. This not relieving his pain, chloral was administered. He went to bed, soon became quiet, and for some hours was left undisturbed. His perfect stillness at length attracting attention, he was found to be dead, and probably had died soon after the administration of the chloral. I have no reason to believe that the medicine was given in larger doses than has been recommended as safe by high authority, nor do I know whether he had taken it for any length of time.

Another case of which I have knowledge was that of a lady, who had undergone a severe surgical operation. As she suffered pain, and was restless, it was determined, in consultation to give chloral by injection, so as to avoid irritating the stomach. A drachm and a half was thrown into the rectum. She at once sank into a state of insensibility, and died in some three hours. An eminent physician of Washington, who was in immediate attendance on the case, Dr. N. S. Lincoln, gave it as his opinion that she died from the effects of chloral.

These cases are, it appears to me, amply sufficient to establish the toxical effects of this powerful agent. It is probable that its poisonous effects are exerted in two ways:

1st. When given in a large dose, and especially when the system may have been charged with it by its previous administration, it at once overwhelms the powers of life, and causes immediate death.

Upon what organ or organs does it exert its deadly effects? It must be either upon the heart or the brain, perhaps on both. It is believed that chloral, entering into the blood, develops chloroform in that fluid, the amount developed being determined not merely by the quantity taken, but by the condition of that fluid. Chloroform, we know, when respired, exerts its influence upon both brain and heart. In the numerous cases in which it has caused death, this result has been produced by its interrupting the circulation.

2d. It appears, when given in small doses and continuously for some time, to induce a form of toxæmia similar to that caused by the continued administration of ergot. Its effects on the fingers of both hands, in the two cases related above, would justify such a belief. It is well known that animals fed on spurred rye suffer gangrene of the extremities.

In one case in which I tested the urine, albumen in notable quantity was detected. This case alone, however, establishes nothing.

Another very interesting and important inquiry is certainly suggested by the foregoing observations, crude as they are.

If chloroform, developed iu the blood from chloral is productive of such disastrous effects, primary and secondary, can the direct inspiration of chloroform be as inocuous as it is thought to be?

The profession are sufficiently aware of the fatal primary effects of chloroform in numerous instances. It has undoubtedly caused death in many cases in which it has been given with every caution in regard to quantity and mode of administration—in cases, too, where there existed no malady of brain or heart to forbid its use. In some instances it has been administered fatally, in which it has been previously treated with good result.

But I would more especially call the attention of the profession to the chronic poisoning of the blood, which I believe results from its free and repeated use.

The writer of this artecle has administered chloroform perhaps as often as any other surgeon in America, both in hospital and private practice, commencing its use from the time of its discovery, and its first application as an anæsthetic. Indeed, I have been constrained to use it in many cases in which my judgment was adverse to its use, for such is the overweening confidence in its effects, that many patients refuse operations except under its influence. But the more I have used chloroform the less has my confidence become in its inocuousness. When I compare the results of my operations performed before anæsthetics were employed with those performed during the last twenty years by the aid of chlorofoım, I am satisfied that unpleasant secondary results were less frequent during the past period than they have been the use of that agent. I allude to secondary hemorrhage, pyæmia, erysipelas, and hospital gangrene.

Whoever will take the trouble to look over the medical journals and retrospects of the last two years, will discover that pyæmia or scepticæmia occupies far more space in surgical records than it did before anæsthetics were so generally employed.

When chloroform is administered during the period of an hour or more, as it frequently is, it undoubtedly enters copiously into the circulation, not only powerfully impressing the brain and heart, but modifying the constitution of the blood and functions of the capillaries. If the effect of chloroform, developed from chloral in the blood, be such as I have shown on the functions of the minute vessels, causing erythema and ulceration in the extreme parts, may we not suppose that the introduction of chloroform more directly into the circulation may promote the occurrence of those results not uncommon before its use?

These suggestions, I trust, will not be regarded as impertinent from one who has practiced surgery for more than half a century, without and with the anæsthetic agents.

I doubt not that, if these remarks are deemed worthy of any notice at all, they will be rejected by the majority of the profession, but I have abiding confidence that their truth will be ultimately acknowledged.—*Boston Medical and Surgical Journal.*

---

